# Patient- and system-related barriers for the earlier diagnosis of colorectal cancer

**DOI:** 10.1186/1471-2296-10-65

**Published:** 2009-09-15

**Authors:** Terry L Wahls, Ika Peleg

**Affiliations:** 1 VA Iowa City Health Care System, Iowa City, Iowa, USA; 2 Center for Research in the Implementation of Innovative Strategies in Practice (CRIISP) VA HSR&D Center of Excellence, Iowa City, Iowa, USA; 3 Division Of General Medicine, Department Of Internal Medicine, University Of Iowa Carver College Of Medicine, Iowa City, Iowa, USA; 4 Division of Gastroenterology, Department of Medicine, University of Oklahoma-Tulsa, Tulsa, Oklahoma, USA

## Abstract

**Background:**

A cohort of colorectal cancer (CRC) patients represents an opportunity to study missed opportunities for earlier diagnosis. Primary objective: To study the epidemiology of diagnostic delays and failures to offer/complete CRC screening. Secondary objective: To identify system- and patient-related factors that may contribute to diagnostic delays or failures to offer/complete CRC screening.

**Methods:**

Setting: Rural Veterans Administration (VA) Healthcare system. Participants: CRC cases diagnosed within the VA between 1/1/2000 and 3/1/2007. Data sources: progress notes, orders, and pathology, laboratory, and imaging results obtained between 1/1/1995 and 12/31/2007. Completed CRC screening was defined as a fecal occult blood test or flexible sigmoidoscopy (both within five years), or colonoscopy (within 10 years); delayed diagnosis was defined as a gap of more than six months between an abnormal test result and evidence of clinician response. A summary abstract of the antecedent clinical care for each patient was created by a certified gastroenterologist (GI), who jointly reviewed and coded the abstracts with a general internist (TW).

**Results:**

The study population consisted of 150 CRC cases that met the inclusion criteria. The mean age was 69.04 (range 35-91); 99 (66%) were diagnosed due to symptoms; 61 cases (46%) had delays associated with system factors; of them, 57 (38% of the total) had delayed responses to abnormal findings. Fifteen of the cases (10%) had prompt symptom evaluations but received no CRC screening; no patient factors were identified as potentially contributing to the failure to screen/offer to screen. In total, 97 (65%) of the cases had missed opportunities for early diagnosis and 57 (38%) had patient factors that likely contributed to the diagnostic delay or apparent failure to screen/offer to screen.

**Conclusion:**

Missed opportunities for earlier CRC diagnosis were frequent. Additional studies of clinical data management, focusing on following up abnormal findings, and offering/completing CRC screening, are needed.

## Background

A growing body of evidence shows that mishandled test results represent a threat to patient safety. In medical records review studies focusing on specific clinical pathology laboratory values (e.g., thyroid stimulating hormone, potassium, hemoglobin a1c, and glucose), 2-18% of cases were found to have clinically significant abnormalities but no evidence of clinician awareness [1-3]. Likewise, studies focusing on specific image types (e.g., mammograms, bone densitometry, and findings of incidental aortic aneurysms) found that 25-40% of cases with clinically significant abnormalities did not have documentation of a clinician response [4-6]. Surveys of primary care providers found that, within the two weeks prior to interview, the majority had seen a patient who had experienced a treatment delay due to missed results [7-10].

A study by Roy [11], found that nearly 1% of hospitalized patients had a significantly abnormal result lost to follow up in the transition between hospital and outpatient care. Numerous studies of diagnostic error utilizing litigation databases have found system-related errors; the most frequent was mishandling of abnormal test results, which was often associated with delays in cancer diagnosis [12-14]. In a cohort study of prostate cancer patients, Nepple et al. [15] found that in more than 16% of the cases, abnormal prostate-specific antigen **(**PSA) results were identified more than six months prior to documented clinician awareness and the diagnosis of prostate cancer.

Various cancers for which patients are commonly screened represent a potential venue in which to study the prevalence of missed results. Colorectal cancer (CRC) is a common cancer [16], and primary care clinicians and the public are aware of recommendations to screen average-risk individuals beginning at age 55. Colonoscopy (CS) is the method of CRC screening preferred by the American Cancer Society and American Gastroenterological Association, while the US Public Health Service Task Force on Preventive Services and the Veterans' Administration (VA) [17] also endorse flexible sigmoidoscopy (FS) and the fecal occult blood test (FOBT) as acceptable modalities for CRC screening. The VA is an excellent venue for studying the prevalence of missed screening opportunities, as it is an integrated healthcare system characterized by a relatively stable patient population and a high rate of CRC screening. The VA's electronic medical record (EMR) system, which is called the Computerised Patient Record System (CPRS), gathers together each patients' clinical notes, along with their clinical, laboratory, imaging, and pathology data [18-20]; thus, the CPRS contains many of the features considered desirable for decreasing the risk of lost test results, and missed screening [21,22].

Our hypothesis was that despite the VA's excellent reputation for patient safety, high rate of documented CRC screening, and state-of-the-art EMR, we would find many patients with missed screening and/or abnormal findings that were lost to follow up, but that could have led to earlier diagnosis of CRC if pursued. The primary examined outcomes were a delay greater than six months between abnormal findings and their subsequent evaluation, and the completion of CRC screening prior to diagnosis. The secondary objectives included the identification of patient- and system-related factors that might be associated with diagnostic delay and/or the lack of CRC screening. We chose to combine missed screening and abnormal findings that were lost to follow up (i.e. missed), as both may lead to the same detrimental outcome.

## Methods

### Setting and Participants

The setting was a VA health care system located in the upper Midwest. This VA provides medical care for over 45,000 veterans annually; primary care is provided in diverse settings across the system, including internal medicine resident continuity care clinics, two hospital-based primary care teams composed of VA providers (physicians, physician assistants, and nurse practitioners), and six smaller community-based outpatient satellite clinics (CBOC), ranging in size from one to nine provider clinics. During the study period, a minority of the primary care was provided in the resident clinics (5%), with the remainder provided in the CBOC (50%) or the hospital-based VA staff clinics (45%). Gastroenterology care was provided at the VA medical center by faculty members and gastroenterology fellows holding dual appointments with the VA and an affiliated medical school. Flexible sigmoidoscopy was provided by primary care clinicians, gastroenterology physicians and fellows, and nurse sigmoidoscopists trained in GI procedures. A minority of colonoscopy screening services were provided by contracted gastroenterologists located in the local communities.

The inclusion criteria were: initial diagnosis of CRC made in the studied VA healthcare system and the availability of clinical notes within CPRS. All CRC cases in the VA tumor registry diagnosed between January 1, 2000 and December 31, 2006 were reviewed. Data were obtained from provider progress notes, nurse notes, appointments, laboratory orders, imaging reports, clinical pathology reports, and anatomic pathology reports.

### Data Collection and Measurement

The collected patient characteristics included: age; the locale in which the patient generally received primary care; comorbid diagnoses; the stage, grade, and location of CRC; and the location of colonic adenomas. Advanced cancer stage was defined as Stage III or Stage IV. Distal location was defined as rectum, sigmoid, or recto-sigmoid. For each patient, a board-certified gastroenterologist reviewed the antecedent medical care records stretching back to the initial contact with the VA or 1995 (whichever came first), and distilled the information into a one-page summary (or clinical abstract). Each clinical abstract included information on CRC screening competed outside the VA, antecedent signs or symptoms, the time between positive findings and subsequent evaluation, any factors that could have contributed to a delay in evaluation, and the reasons for CRC diagnosis.

The included CRC screening modalities were FOBT, barium enema (BE), flexible sigmoidoscopy (FS), and colonoscopy (CS). Completed screening was defined as documented completion of CRC screening outside the VA in the five years (10 years for CS) prior to CRC diagnosis, or the availability of these results in CPRS (i.e. completion of tests within the VA) within this same period. Issues and circumstances that were believed to potentially contribute to diagnostic delay were classified as either patient- or system-related factors (Table 1). Delay was defined as a gap of more than six months between the detection of abnormal findings and completion of CRC evaluation. The abovementioned patient- and system-related factors were also collected from patients for whom no prior CRC screening was documented. Completed evaluation was defined as a clinical explanation of the abnormal findings. The clinical abstracts were reviewed by TW and IP and coded through consensus.

**Table 1:** Patient- and system-related factors with diagnostic delay

**Patient Factors**
Frequent appointment no-shows
Frequent appointment cancellations
Declined evaluation
Medical comorbidity
Poor-quality information from patient history
Reference to patient noncompliance
Significant psychiatric diagnosis
Homelessness

**System factors**

Scheduling delay for colonoscopy
Incorrect Interpretation of results
Judgment error by clinician
Communication failure of clinician
Inexperience of clinician

**Abnormal findings likely lost to follow up**

Outside records not obtained
Weight (wt) loss not evaluated
Anemia not evaluated
FOBT request
Positive FOBT
Abnormal flexible sigmoidoscopy, colonoscopy or polyp biopsy results
Abnormal barium enema or CAT scan results
Lesion missed by colonoscopy or flexible sigmoidoscopy
Lesion missed by barium enema

Approval for the study was obtained from the Institutional Review Board of the University of Iowa and the Iowa City VAMC.

## Results

### Participant Characteristics

Of 156 patients newly diagnosed with CRC during the study period, 150 met the study inclusion criteria. The mean age at diagnosis was 69.04 years. Sixty-seven (44%) cases had late stage CRC, and 71 (47%) had a distal CRC location. Fifty-one cases were found through screening. FOBT was used as the initial screening modality in 14 cases; other screening modalities included FS (26 cases), and CS (9 cases). In two patients, CRC was detected by surveillance colonoscopy performed because of a personal history of adenomatous colonic polyps. Eighteen cases were diagnosed as part of the patient's initial contact with the VA, two because of a positive CRC screening completed as part of the initial health-maintenance screening, and 16 because of symptoms present at the time of initial contact. Ninety-nine cases were found due to symptoms; these included 43 cases (29%) with anemia, and 22 (15%) with either rectal bleeding or melena. For more detail, see Table 2.

**Table 2:** Reasons for diagnosis of CRC

	**All CRC cases (N = 150)**	**Veterans diagnosed as part of initial presentation to VA (N = 18)**
**Initial reason for diagnosis**		
Positive fecal occult blood test	14	0
Positive flexible sigmoidoscopy	26	2
Positive colonoscopy	9	0
Hx polyp	2	0
**Total found through screening**	**51**	**2**
Rectal bleeding or melena	22	5
Anemia	43	4
Pain	13	0
Wt loss	10	4
Abnormal CT scan	4	0
Metastatic disease	8	3
**Total found through investigation of symptoms**	**99**	**16**

### CRC Screening

Ninety-eight patients (65%) had CRC screening documented prior to diagnosis; 48 (32%) had received FOBT, 31 (20.3%) had received FS, and 19 (12.4%) had received CS. Two cases were under 50 years old, and three cases were over 80 years old. Forty cases that were age-appropriate for screening lacked documentation of CRC screening being completed or offered. The medical records for four of the un-screened cases had documentation of an offer to screen; three also had documentation that the patient had declined screening. From among the cases, 19 and 24 had patient- and system-related factors, respectively, that were believed to potentially contribute to either the absence of screening or diagnostic delay. Twenty-one patients were age-appropriate for screening and had received prior ongoing, longitudinal care from the VA; of them, 16 had no documentation of CRC screening being offered or completed. Of the 71 cases with distally located CRC, 44 had no documentation of offered/completed CRC screening. Twenty-six of the cases without diagnostic delay lacked documentation of CRC screening prior to diagnosis. For more detail, see Table 3.

**Table 3:** Documentation of completed CRC screening in the medical record

**Total cases with no documentation of CRC screening completed prior to diagnosis**	**45**
No Screen: Age < 50 y/o	2
No Screen: Age > 80 y/o	3
No Screen: Age ≥50 and ≤80 y/o	40
Documented offer to screen + patient declination of screening	3
Documented offer to screen but lost to follow up	1
Patient factors identified (N = 19 cases)	19
Frequent no-shows/cancellations	7
Declined therapy/evaluation	11
Comorbidity	7
Psychiatric diagnosis	6
Homelessness	3
System factors identified (N = 24 cases)	24
Scheduling delays	3
Abnormal findings lost to follow up	21
Wt loss	3
Anemia	13
FBOT request	1
Judgment error by clinician	3
No screening despite appropriate age, no patient factors identified, and ongoing antecedent care with the VA	21

### Cases with Delays in the Evaluation of Abnormal Findings

**Figure 1:**
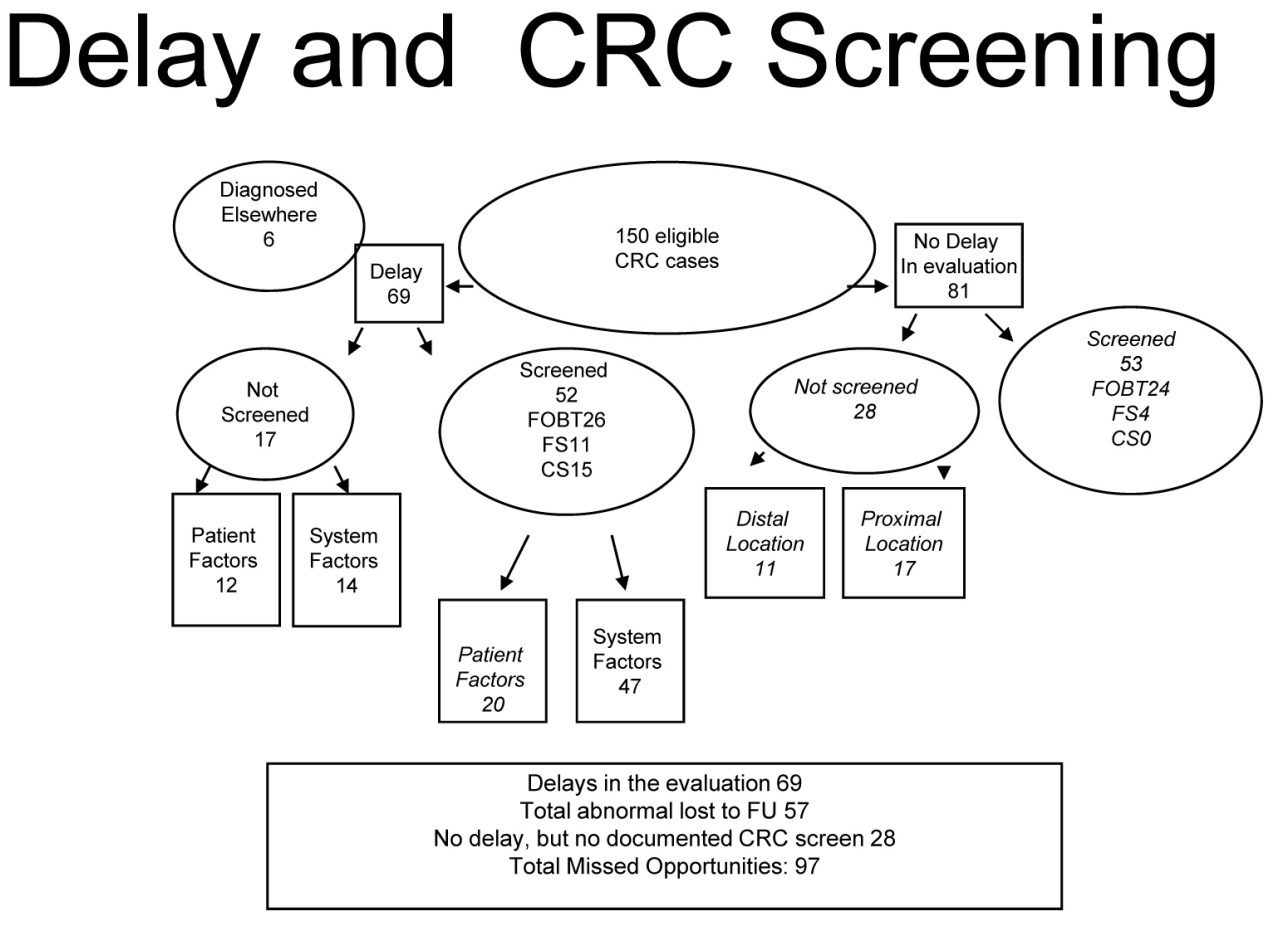
**Flow chart summarizing the presence of prior CRC screening and apparent diagnostic delay**.

In 69 cases, more than six months elapsed between the abnormal test results and diagnosis of CRC. A total of 212 individual system factors were identified in 61 cases (mean 3.8 per case, range 0-8). A total of 56 individual patient factors were identified in 32 cases (mean 3.1 per case, range 0-10). For more detail, see Figure 1. Both patient- and system-related factors were identified in 25 cases, and two or more factors were identified in 63 cases. For more detail, see Tables 4 and 5.

**Table 4:** Delays and frequency of possible contributory factors

	**System factors**	**Delay obtaining colonoscopy due to scheduling issues**	**Abnormal findings lost to FU per case**	**Patient factors**	**All reported factors**
Total number of factors reported	212	6	101	56	268
Mean number of factors reported per case	3.1	0.09	1.5	0.8	3.9

**Table 5:** Factors contributing to diagnostic delay

**Factors that may have contributed to the development of delay** **(N = 69 cases)**	**Number of factors identified**
Patient factors (N = 32 cases)	56
	
Frequent appointment no-shows	9
Patient declined evaluation	16
Comorbidity	18
Frequent appointment cancellations	4
Poor information from patient history	0
Noncompliance	0
Major psychiatric diagnosis	8
Homelessness	1
System factors (N = 61 cases)	212
Delay in CS scheduling due to backlog in VA	4
CS or FS missed the lesion(s)	10
Barium enema missed the lesion(s)	1
Incorrect interpretation of findings	1
Error in clinical judgment	1
Communication breakdown	1
Inexperience of clinician	0
Abnormal findings likely lost to follow up (N = 57 cases)	101
Review/obtain outside records	8
Wt loss	7
Anemia	41

	
Positive family history	2
FOBT request	4
Positive FOBT	22
Abnormal colon polyp biopsy	3
Abnormal imaging	2
Hematochezia	3
Abnormal findings lost to follow up with no patient factors identified	33

**Figure 2:**
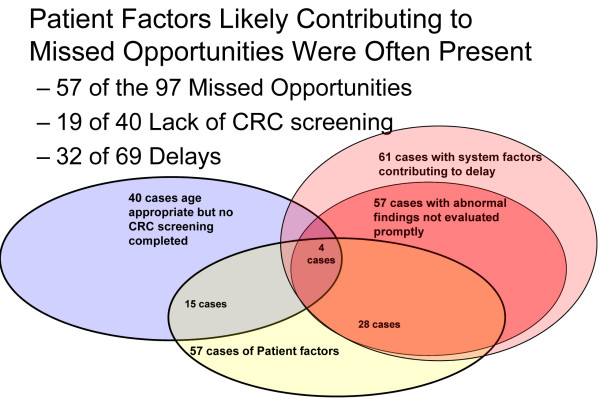
**Distribution of patient factors that appear to contribute to diagnostic delay or the absence of CRC screening**.

In 11 cases, a diagnostic or imaging procedure appeared to have missed an existing lesion (barium enema in one case, CS or FS in 10 cases). A total of 101 different abnormal findings in 57 cases failed to receive a completed evaluation within six months. The two most frequent abnormal findings that failed to receive a completed evaluation within six months were anemia (41 cases) and positive FOBT (22 cases). Thirty-two of the cases with delayed evaluation of abnormal findings were found to have patient factors that may have contributed to the development of delay. These included patient declination of evaluation (16 cases), frequent patient-initiated appointment cancellations (4 cases) and no-shows (9 cases), comorbid medical conditions (16 cases), comorbid psychiatric diagnoses (8 cases), and homelessness (1 case). Thirty-three cases with delays had no identified patient factors. For more detail, see Figure 2.

## Discussion

In nearly two thirds of the CRC cases examined in the present study, an opportunity for earlier diagnosis had been missed. Half of the distally located cancer cases had no prior CRC screen, and a third of the studied CRC cases lacked documentation of CRC screening having been offered or completed prior to diagnosis. Delays associated with system errors were noted in over a third of all CRC cases, with delayed response to anemia and positive FOBT comprising the most common errors. In addition, patient factors were identified in half of the cases with missed opportunities for earlier diagnosis. These data, though troubling, are consistent with the growing body of literature on diagnostic error and missed results. The present study found that while patient factors often contributed to delays, the majority of delayed diagnoses suffered from system factors, particularly lost follow up of abnormal findings. This relatively frequent loss of abnormal findings to follow up is consistent with several lines of evidence in the literature which have shown that delays are most often the result of an accumulation of multiple errors [12-14];

System factors were identified in two-thirds of the cases with delays; the vast majority involved failures to promptly evaluate abnormal findings. Although patient factors were often present, only eight cases involved underlying medical or psychiatric co-morbidities. Most delays had multiple system factors, including failure to screen. Although all of the relevant system factors should be addressed, the following focuses on issues related to the management and presentation of clinical data.

Because screening is recommended for colorectal, breast, cervical, and prostate cancer, a large volume of cancer screening tests may be requested and completed annually within the practice of a given primary care practitioner. Nationally, these four cancers together accounted for an estimated 532,000 new diagnoses and 124,000 deaths in 2007 [16]. Although public acceptance of cancer screening is relatively high, the rate of CRC screening remains below that for breast, cervical, and prostate cancer [24-27]. Patients often report they have not been counseled to seek CRC screening [28-30], even though primary care practitioners have begun to face malpractice litigation for diagnostic delay when cancer is diagnosed in the absence of a prior screen [13,31].

Hundreds of thousands (if not millions) of cancer screening diagnostics are requested and completed each year. With the typical primary care provider ordering over a thousand tests each week, providers can easily be overwhelmed by the volume of data that must be reviewed [22], increasing the risk of individual results being missed (i.e. lost to follow up). Although EMRs can efficiently deliver results to providers, this does not guarantee that the provider will interpret the findings correctly and respond appropriately [5,22]. The psychology literature demonstrates that as work increases and alarm sensitivity declines, 90% of individuals will produce progressively lower-quality work and their responsiveness to alarms will decline [32,33]. Furthermore, numerous studies have indicated that providers often ignore drug alert warnings [34-39] and even abnormal test results [39] within their EMR.

It is therefore necessary to improve the management and presentation of clinical data to providers. Lessons can be learned from the fields of industrial engineering and aviation [39,40], which have sought to redesign processes, data presentation, and decision making to ensure that the data volume is consistent with human limitations [41]. It could be helpful to develop computer algorithms capable of filtering data for abnormal results and recognizing when a positive finding has already been evaluated and is stable (and therefore is not a concern), with the goal of presenting clinicians with only information that requires a clinical response [10,41].

One solution that has been suggested to deal with data management issues is the direct notification of patients by the laboratory service upon completion of CRC screening [42]. While a slim majority of patients expressed a desire for direct notification, many physicians are uncomfortable with this solution as it can lead to increased patient anxiety and telephone calls, and additional non-compensated work for the clinicians [5]. However, when patients are not given their test results, they tend to be less motivated, experience lower levels of therapeutic adherence, and may have poorer outcomes [4,5,43,44].

## Limitations

This study has two main limitations. First, VA patients can also receive healthcare services in the community. It is therefore possible that evaluations of abnormal findings and CRC screenings were completed in the community without being included in the VA EMR. However, we reviewed all primary care and GI progress, nurse, and procedure notes, which often explicitly stated whether screening had (or had not) occurred in the community. Furthermore, the medico-legal system places higher value on care that is documented in the medical record [43], and the present findings are consistent with those from prior studies documenting that 10% of CRC had been missed by prior imaging studies [45] and more than 10% of positive FOBTs receive inadequate follow up [46]. The second limitation is that this study was completed solely in the VA, and will thus require replication in other healthcare systems. However, the present results are consistent with those from a cohort of men diagnosed with prostate cancer, in which one out of five cases showed delays of more than six months between an abnormal PSA test result and documented clinician awareness [23]. Furthermore, multiple studies have shown that abnormal test results lost to follow up (i.e. missed results) occur in diverse settings (e.g., academic, private, VA, hospital, and ambulatory settings), involve a variety of staff types (e.g., trainees, staff physicians, and mid-level providers), and across multiple types of diagnostic studies [1-4,11,47]. As such, the present results add to the growing body of evidence suggesting that problems with consistent management of patient test result data contribute to diagnostic error and unnecessary diagnostic delays more often than is generally appreciated.

## Conclusion

In a cohort of patients diagnosed with CRC, opportunities for earlier diagnosis were frequently missed. Contributory patient factors were identified in only half of the cases with delayed or absent CRC screening. Although clinicians were using an advanced EMR, this was insufficient to ensure CRC screening or prevent delays in the evaluation of abnormal findings. Additional studies are warranted to examine clinician management of abnormal laboratory test results and failure to document the offer and/or completion of CRC screening.

## Competing interests

The views expressed in this article are those of the authors and do not necessarily reflect the policy or position of the Department of Veterans Affairs.

## Authors' contributions

TW and IP participated in the study concept, design, acquisition of data, analysis and interpretation of data, drafting of manuscript and critical revision of the manuscript for intellectual content. IP completed the statistical analyses. TW provided administrative and clerical support. All authors read and approved the final manuscript.

## Pre-publication history

The pre-publication history for this paper can be accessed here:


